# Internet-Delivered Cognitive Behavioral Therapy for Fathers With Postnatal Depression: Randomized Controlled Trial of DadBooster

**DOI:** 10.2196/94541

**Published:** 2026-09-01

**Authors:** Charlene Holt, Jennifer Ericksen, Alan W Gemmill, Andre L Rodrigues, Jeannette Milgrom

**Affiliations:** 1 Parent-Infant Research Institute, Heidelberg Repatriation Hospital, 300 Waterdale Rd, Heidelberg Heights, Victoria, 3081, Australia, 61 394964496; 2 Melbourne School of Psychological Sciences, The University of Melbourne, Parkville, Victoria, Australia

**Keywords:** father, paternal, postnatal depression, postpartum depression, depression, cognitive behavioral therapy, treatment, therapy, digital, online, eHealth, randomized controlled trial

## Abstract

**Background:**

Up to 1 in 10 fathers experience postnatal depression. Fathers are less likely to seek help and receive adequate treatment than depressed mothers. Digital treatments hold significant potential for engaging and supporting fathers, but no effective program exists.

**Objective:**

The aim of the study is to evaluate the efficacy of an online cognitive behavioral therapy program, DadBooster, for treating postnatal depression in fathers.

**Methods:**

A parallel, 2-group randomized controlled trial (N=50) was conducted to test the superiority of DadBooster over waitlist control who received routine care. Randomization was computer-generated, with allocation concealment maintained through central, computer-automated administration. Participating fathers were aged 18 years or older, had a baby younger than 12 months, had a depression diagnosis using the Quick Structured Clinical Interview for DSM-5 (QuickSCID-5), were not receiving depression treatment, and did not meet criteria for other mental health disorders. They were recruited Australia-wide and completed questionnaires online and assessments by telephone. Primary outcomes were depression symptom severity (Depression Anxiety Stress Scales-21 [DASS-21]) and depressive episode remission (QuickSCID-5) 12 weeks after enrollment. Participants were not blinded. Assessments (QuickSCID-5) and data analysis were conducted blind to allocation.

**Results:**

Participants (N=50) were randomized (DadBooster: n=25; waitlist control: n=25) and analyzed as assigned; the trial is complete. Mean depression symptoms (DASS-21) at 12 weeks were significantly lower for DadBooster than waitlist control (adjusted mean difference −7.26, 95% CI −11.34 to −3.18; *F*_1,47_=12.81; *P*<.001; η_p_^2^=0.21); average scores reduced by 72% (compared to 40% in waitlist control) and dropped to the “normal” range at 12 weeks. Of the DadBooster participants assessed, 2 of 23 (9%) still met diagnostic criteria (QuickSCID-5) at 12 weeks compared with 7 of 24 (29%) waitlist controls; not significant (Fisher exact test: *P*=.14). Significant differences were found for stress (adjusted mean difference −6.55, 95% CI −11.01 to −2.09; *F*_1,47_=8.73; *P*=.005; η_p_^2^=0.16), parenting self-efficacy (adjusted mean difference 4.06, 95% CI 1.16-6.95; *F*_1,47_=7.93; *P*=.007; η_p_^2^=0.14), and negative automatic thoughts (adjusted mean difference −18.10, 95% CI −29.79 to −6.42; *F*_1,47_=9.71; *P*=.003; η_p_^2^=0.17). Participants engaged well with the intervention—80% (20/25) visited 4 or more sessions—and attrition was low. No adverse events were identified.

**Conclusions:**

This randomized controlled trial demonstrates the efficacy of DadBooster, the first online treatment targeting clinically diagnosed postnatal depression in fathers, addressing a critical gap in the treatment of postnatal depression, an area that has until recently been primarily focused on mothers. By providing evidence for an intervention specifically designed for fathers with potential for broader scalability, this study advances the limited literature on treatment approaches for postnatal depression in fathers. Given its accessibility, privacy, and convenience, DadBooster is a promising and potentially impactful intervention for supporting fathers.

## Introduction

### Postnatal Depression in Fathers

At least 1 in 10 fathers with a new baby experiences depression [1,2]. Prevalence figures vary according to population characteristics, measure used (screening vs diagnostic), and whether point or period prevalence is reported. Postnatal depression in fathers is typically defined in the research literature as depression occurring within the first year following the birth of a child [3]. Diagnosis is based on the same symptoms as depression in the general population. Symptoms include lowered mood, loss of interest or enjoyment, sleeping difficulties, changes in appetite and weight, and feelings of worthlessness [4]. Depressed fathers may experience additional symptoms that are specific to men including anger and irritability [5] and may be more likely to engage in risk-taking, substance abuse, and family violence than nondepressed fathers [6,7]. Suicide risk in men is 5%‐6% during the perinatal period [8,9]. Depression is also often accompanied by comorbid anxiety [10].

Risk factors for postnatal depression in fathers include a history of mental health difficulties, partner’s depression, and a range of psychosocial factors including relationship dissatisfaction, low parenting self-efficacy, financial difficulties, and low social support [8,10-12]. A father’s depression may adversely affect their partner’s mental health and vice versa [13] and is also associated with poorer developmental outcomes for the baby [14]. A recent systematic review [14] highlighted the perinatal period as a critical window for infant development, emphasizing the need for interventions to support perinatal mental health in fathers, given its influence as a modifiable determinant of child outcomes. Postnatal depression in fathers also imposes a substantial economic burden on the Australian health care system [15]. Together, these findings highlight that postnatal depression in fathers is a significant public health concern, with important implications for families, health care systems, and child development.

### Engaging Fathers in Treatment

Despite these far-reaching effects, few depressed new fathers are identified through current health care systems that are primarily focused on the well-being of mothers and babies [16]. Around 45% of fathers report that they are not aware of postnatal depression in fathers [15]. Fathers are also less likely to seek help or engage with effective treatment compared with mothers [17,18]. This parallels findings in the general population that men are less likely to acknowledge depressive symptoms and access mental health supports than depressed women [19,20]. Endorsement of traditional masculine norms has been implicated in men’s reduced recognition of distress and reluctance to seek support [20,21]. It has been suggested that gender-sensitive services and interventions are needed to optimize men’s help-seeking and engagement with treatment [19,20]. Given this, intervention approaches that minimize barriers related to stigma, accessibility, and engagement may be particularly important for fathers. However, there remains a relative lack of evidence-based treatments specifically targeting postnatal depression in fathers, particularly compared to those available for mothers [22]. Despite its prevalence and impact, effective and accessible treatment options for fathers remain limited, highlighting a critical gap in perinatal mental health care.

### Digital Treatment Programs

Digital treatments hold significant potential for engaging and supporting fathers [17,23]. They have the benefits of being more accessible than conventional face-to-face treatments, can be accessed privately, without a need for referral, and are typically available anywhere and at any time, reducing some of the access barriers of face-to-face treatments [16]. These features may be particularly advantageous for fathers, given their lower likelihood of help-seeking and the potential to reduce barriers related to stigma and accessibility. Consistent with this, a recent meta-analysis [24] identified significantly lower initiation rates for face-to-face treatment compared to digital treatment within clinical populations that included men. It was postulated that delays in commencing conventional treatment may negatively impact motivation and engagement.

Cognitive behavioral therapy (CBT) is a first-line, evidence-based psychological treatment for depression in both men and women [25,26]. There is also good evidence for digital CBT programs for depression in the general population [27] and for maternal postnatal depression specifically [28], with results showing online treatments to be as effective as traditional face-to-face CBT. Given these findings, digital CBT may also have strong potential for fathers, particularly in addressing known barriers to engagement with conventional services.

However, a recent review found that there were no specialized digital treatments for postnatal depression in fathers [22]. Despite growing research attention on paternal perinatal mental health, there is a lag in the development of tailored intervention approaches.

Since this review, there has been only 1 published randomized controlled trial (RCT) evaluating a digital treatment for postnatal depression in fathers [29]. This study evaluated a mindfulness-based CBT mobile app for fathers who self-reported moderate symptoms of depression, anxiety, or stress during the perinatal period. No significant differences were found between the mindfulness app and app-based mood monitoring (control condition), possibly due to low adherence with the intervention and moderate attrition over the study.

### The DadBooster Program

To address the lack of digital treatments for postnatal depression in fathers, this study developed and evaluated a treatment program tailored for fathers, DadBooster. The DadBooster program uses the core CBT components and digital architecture of our postnatal depression treatment program for mothers, MumMoodBooster, that is now delivered at scale in Australia. MumMoodBooster was evaluated in RCTs and shown to be highly effective [28,30,31]. Tailoring to the needs of fathers was informed by consumer consultation and previous work by Domoney et al [32], who gathered expert consensus on key components of CBT for postnatal depression in fathers. These adaptations included father-focused imagery and videos, tailored language and examples reflecting common paternal experiences, a design and visual style informed by father preferences, and a more concise, less text-heavy presentation of content. DadBooster was developed specifically for fathers with a diagnosed depressive disorder.

### Objectives

This study aimed to evaluate the efficacy of the DadBooster online program in reducing symptoms of postnatal depression (both severity and diagnostic status) in fathers compared to routine care in an RCT. Secondary aims were to examine effects on anxiety and stress symptoms, cognitive-behavioral skills (negative thinking and behavioral activation), partner relationship quality, and parenting self-efficacy.

We hypothesized that fathers allocated to the DadBooster condition would experience a reduction in depression symptom severity of at least 50%—significantly greater than reductions observed in the waitlist control (routine care) condition. We further hypothesized that a significantly smaller proportion of fathers would meet clinical diagnostic criteria for depression at follow-up compared to the waitlist control condition. Secondary hypotheses were that, relative to controls, fathers in the DadBooster condition would report lower levels of anxiety and stress, reduced negative thinking, greater behavioral activation, improved partner relationship quality, and higher parenting self-efficacy.

## Methods

### Program Development

The DadBooster program was designed to appeal to and engage fathers, recognizing that fathers exhibit and experience emotional distress in ways specific to men. Development started with the key CBT content of our effective MumMoodBooster treatment program (for women with postnatal depression) that had adapted CBT to be relevant in the postnatal period. Consumer feedback (see Patient and Public Involvement section) from fathers with young children informed the development of DadBooster, designed for fathers experiencing depression in the first year after the birth of a baby. The program development team comprised clinicians with expertise in postnatal depression in fathers, along with researchers in the field who are also fathers.

Following consumer input and identification of necessary changes, the prototype was refined by the program development team. Beta-testing was carried out, and the required modifications were made, resulting in the first version of DadBooster for evaluation in the RCT (see Intervention—DadBooster section).

### Patient and Public Involvement

During the program development phase, consumer input was obtained via interviews with 6 fathers who provided general feedback on the prototype program, including their preferences for resources for fathers, and specifically on the logo, name of the program, colors, images, and the way information is presented. The prototype program was adapted based on this feedback. An additional 13 fathers then provided further feedback via online survey on the look and feel of the prototype program and associated resources, images used, colors, logo, and name.

During the RCT phase, participants (fathers with depression) provided feedback on the program through follow-up questionnaires. This feedback informed minor refinements to the DadBooster intervention during the trial, such as addressing technical issues and usability problems identified by participants. Patients or the public were not involved in the study design, outcome selection, or paper preparation.

### RCT

#### Trial Design

This was a parallel, 2-group RCT with a 1:1 allocation ratio, in which individual participants were randomized to test the superiority of DadBooster over a waitlist control for fathers with diagnosed depression. The primary outcomes were severity of symptoms of depression and remission from the depressive episode at 12 weeks after enrollment. The trial was prospectively registered with the Australian and New Zealand Clinical Trials Registry on July 22, 2022 (ACTRN12622001029785). The first participant was enrolled on August 30, 2022; recruitment continued until July 2024. A single deviation from the registered protocol is described in the Statistical Methods section. In addition, a clarification regarding blinding procedures is provided in the Blinding section, as the trial registration did not fully capture assessor and analyst blinding. The trial is reported in accordance with the CONSORT (Consolidated Standards of Reporting Trials) 2025 statement and the CONSORT-eHEALTH (Consolidated Standards of Reporting Trials of Electronic and Mobile Health Applications and Online Telehealth) guidelines for web-based and mobile health interventions (Checklists 1 and 2) [33,34].

#### Trial Setting

The trial was conducted in Australia, with participants recruited nationally across rural and metropolitan areas. Participants were recruited directly from the community via online methods, as well as through referral pathways from health professionals and services. All study procedures and intervention delivery were conducted remotely (primarily online with additional telephone contact) by the Parent-Infant Research Institute. The trial was a single-site study.

#### Participants

Marketing focused on social media campaigns, as well as promotion to health professionals and services (eg, maternal and child health nurses, early parenting centers, and perinatal mental health services) via information sessions, newsletter articles, and conference displays. Health professionals were encouraged to refer their clients to the project. Interested individuals followed links or QR codes from posters, postcards, or social media to obtain further information and to begin the screening process by visiting the secure project recruitment website. Informed consent was obtained from all trial participants.

### Eligibility

#### Inclusion and Exclusion Criteria

Inclusion criteria were (1) fathers aged 18 years and older, (2) infants aged less than 1 year, (3) Australian residency, (4) English speaking, (5) no current treatment for depression (medication or therapy) to ensure effects could be attributed to the DadBooster intervention, (6) presence of depressive symptoms based on the Patient Health Questionnaire-9 (PHQ-9 ≥9) [35], and (7) diagnosis of current major depressive disorder or depressive disorder with insufficient symptoms.

Exclusion criteria were (1) Post-Traumatic Stress Disorder using the Primary Care PTSD screen for DSM-5 [36], (2) psychotic symptoms using the DSM-5 Self-Rated Level 1 Cross-Cutting Symptom Measure—Adult Psychosis domain [37], (3) moderate to high suicide risk based on PHQ-9 item 9 score, (4) current substance use disorder, and (5) current or past bipolar disorder.

There were 3 sequential steps in determining eligibility for participation in the study: an online screening survey, an intake phone call, and a clinical assessment.

#### Online Screening

Initial screening assessed inclusion criteria 1‐5. Individuals who satisfied screening criteria were emailed a link to read and sign a participant information and consent form.

#### Intake Call

Consenting men were telephoned by a psychologist for an intake call. The study was explained, and further screening and risk assessment were conducted. Men were screened for inclusion criterion 6 and exclusion criteria 1‐3. This call included a clinical risk assessment following a structured risk protocol. Those deemed to be at moderate to high risk for suicide were excluded and referred to receive immediate crisis attention (n=2). A clinical assessment was scheduled with those who satisfied eligibility criteria assessed as part of this intake call.

#### Clinical Assessment

Following the intake call, eligible consenting fathers were assessed by a psychologist using the Quick Structured Clinical Interview for DSM-5 (QuickSCID-5 [38]) by telephone. Inclusion criterion 7 was assessed using the QuickSCID-5 assessment, as well as exclusion criteria 4 and 5. Results of the QuickSCID-5 were discussed with each prospective participant, and all were asked to nominate a general practitioner (previous, current, or newly identified) to whom the psychologist could send notification of their diagnosis, study participation, and treatment allocation.

### Treatment Conditions

#### Intervention—DadBooster

DadBooster is a self-guided, web-based CBT program that was designed to be closely comparable in content to face-to-face treatment for depression and completed by participants at home. The program was supported by automated, one-way text messages sent twice weekly (11 messages in total over 6 weeks) to encourage engagement and introduce upcoming session content. The program was designed to treat depressive symptoms, with additional components included to support related domains such as parenting and couple relationship functioning.

DadBooster used tunnel information architecture to guide users through 6 sequential weekly sessions: (S1) getting started, (S2) managing your mood, (S3) increasing your pleasant activities, (S4) managing negative thoughts, (S5) increasing positive thoughts, and (S6) planning for the future. Sessions incorporated core CBT components, including psychoeducation about depression, mood monitoring, relaxation strategies, behavioral activation, cognitive restructuring, strategies to increase positive thinking, and relapse prevention. A scaffold approach was used to build upon content shown in previous sessions.

Each session was designed to be completed in approximately 20‐30 minutes and included text, audios, videos, and interactive activities to address the user’s individual issues and promote engagement. Between-session practice activities (“homework”) were recommended in every session. Feedback and reinforcement were provided within the program to support engagement and completion of homework activities. This was delivered via prerecorded session introduction videos presented by the program host, with content tailored based on the user’s activity completion.

The program included interactive charts that displayed user-entered data and depicted therapeutic concepts in CBT, such as the functional relationship between mood and pleasant activities. The user could personalize the program by changing the images that appear on each page to their own personal photos. Additional informational articles written specifically for the program were included in the DadBooster library, which users could access at any time. Topics covered were problem-solving strategies, stress management, time management, communication skills, information on enhancing relationships with partner and baby, getting support, and tips for caring for a baby. These articles were designed to directly target secondary outcome domains, particularly parenting and couple relationship quality, and to provide fathers with additional coping strategies.

Participants were encouraged to invite their partner to access a standalone partner support website, developed specifically for partners, with a separate user login. The partner support website included information for partners about caring for themselves and their baby and resources if they were struggling with postnatal depression themselves, while also providing information about paternal depression and what their partner may be experiencing.

The program included a printable workbook summarizing the activities completed in the program, and participants were encouraged to print it after session 6 as a hard copy reference. DadBooster was optimized for use on both desktop and mobile devices, and participants retained access for at least 6 months from enrollment. Minor refinements to the program were made during the trial to address technical issues and usability concerns identified by participants, without altering the core therapeutic content.

**Figure 1.**
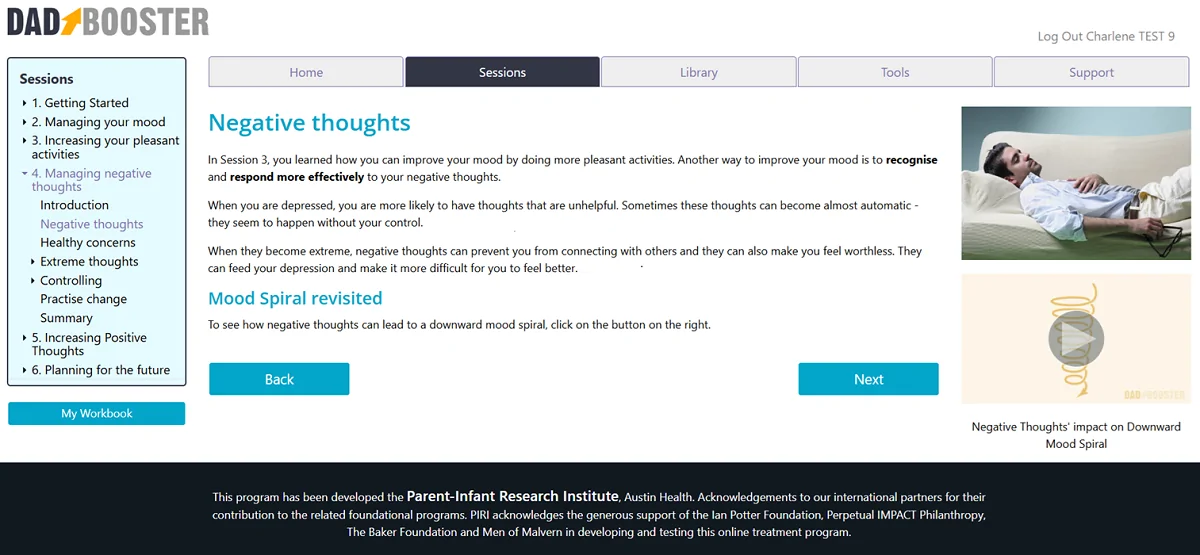
Screenshot from session 4 of the DadBooster web-based cognitive behavioral therapy program for fathers with postnatal depression.

Adherence was measured unobtrusively using website analytics and database flags (see Intervention and Comparator Delivery section). A screenshot from session 4 is presented in Figure 1. The program is available from the Parent-Infant Research Institute [39].

#### Comparator—Waitlist Control

As in the DadBooster condition, a letter was sent to the participant’s general practitioner (GP). General practitioners were notified that their patient met diagnostic criteria for depression, and they were encouraged to consult with their patient regarding their mental health care needs. Support and/or referral to other services then occurred as necessary, as would normally happen where specialized programs are not available. Therefore, routine care varied at the discretion of each general practitioner and included a mix of interventions or supports. Following completion of the 3-month postenrollment follow-up, participants in this group were provided with full access to DadBooster.

### Outcomes

Measures were administered via online questionnaire or by telephone at 3 main time points: baseline, 9 weeks after enrollment, and 12 weeks after enrollment. Demographic data (age, education, income, number of children, marital status, and location of birth) were gathered both during screening telephone calls and online baseline questionnaires.

#### Primary Outcome Measures

At baseline and at 12 weeks after enrollment, severity of depressive symptoms was measured online using the Depression Anxiety Stress Scales-21 (DASS-21 [40]). The DASS-21 is a 21-item, 4-point Likert-type scale. Each of the subscales (depression, anxiety, and stress) has a maximum score of 42. The Depression Anxiety Stress Scale (DASS) manual provides recommended cutoffs for rating the severity of depression as normal (0‐9), mild (10-13), moderate (14-20), severe (21-27), or extremely severe (28+).

At baseline and at 12 weeks after enrollment, psychologists trained to administer the QuickSCID-5 [38] conducted assessments by telephone to determine a *Diagnostic and Statistical Manual of Mental Disorders-5* (DSM-5) diagnosis of current major depressive disorder or depressive disorder with insufficient symptoms. These psychologists were blind to treatment allocation at the 12-week follow-up time point.

#### Secondary Outcome Measures

##### Depressive Symptom Trajectory

Participants’ depressive symptoms and safety were monitored using PHQ-9 [35], which was completed online at baseline and 2, 4, 9, and 12 weeks after enrollment.

##### Anxiety and Stress Symptom Severity

Participants’ anxiety and stress symptom severity were measured at baseline and 12 weeks after enrollment using the anxiety and stress scales of the DASS-21 [40]. Recommended cutoffs for rating the severity of anxiety are normal (0‐7), mild (8-9), moderate (10-14), severe (15-19), or extremely severe (20+). For stress: normal (0‐14), mild (15-18), moderate (19-25), severe (26-33), or extremely severe (34+).

##### Negative Thinking

At baseline and 12 weeks after enrollment, participants completed the Automatic Thoughts Questionnaire [41], which includes 30 items assessing the occurrence of negative thoughts over the previous week. Participants rated their agreement with a series of statements (eg, “I’m a failure”) using a scale from 1=not at all to 5=all of the time. The maximum score is 150.

##### Behavioral Activation

At baseline and 12 weeks after enrollment, participants completed the Behavioral Activation for Depression Scale [42], which includes 25 items measuring changes in behavior. Participants rated their agreement with a series of statements (eg, “I made good decisions about what type of activities and/or situations I put myself in”) on a scale from 0=not at all to 6=all the time, with a maximum total score for the scale of 150.

##### Relationship With Partner

At baseline and 12 weeks after enrollment, participants’ general satisfaction in their relationships with their partners was assessed using the Dyadic Adjustment Scale-7 (DAS-7 [43]), which was calculated as the sum of all items. The maximum score is 36. Scores below 24 are considered “distressed.”

##### Self-Efficacy in Parenting Role

At baseline and 12 weeks after enrollment, the self-efficacy subscale of the Parenting Sense of Competence Scale [44] was used to assess participants’ self-perception of competence and capability in the parenting role. Seven statements (eg, “Being a parent is manageable, and any problems easily solved”) were rated from 1=strongly disagree to 6=strongly agree) with a maximum score of 42.

##### Engagement in DadBooster Program

Program use was measured unobtrusively using website analytic tools and database flags that recorded the number and duration of visits to the program and program sessions or content accessed.

##### Program Feedback

At 9 weeks after enrollment, participants completed a feedback form that asked “Overall, how helpful did you find the DadBooster program” with response options 1=not at all helpful, 2=somewhat helpful, 3=moderately helpful, and 4=very helpful. They were also presented with a list of program features and asked to rate how satisfied they were with each, with response options 1=not at all satisfied, 2=somewhat satisfied, 3=moderately satisfied, and 4=very satisfied. Participants were asked which of the following topics they would like a library article on: feeling angry and irritable, alcohol and substance use, men’s attitudes to help-seeking, and other. Feedback was collected at 9 weeks, as most participants were expected to have finished the intervention at this time, allowing for timely evaluation of acceptability and satisfaction. At 12 weeks after enrollment, participants completed an adaptation of Brooke’s [45] System Usability Scale to rate the usability of the program.

##### Use of Other Supports or Treatments

At 12 weeks after enrollment, participants were asked to indicate from a list which other support services and treatments they accessed during the study with the option to add others not listed.

### Harms

Safety was monitored throughout the study, focusing on worsening depressive symptoms and suicide risk. These were assessed via the PHQ-9 at baseline and 2, 4, 9, and 12 weeks after enrollment. Predefined thresholds (≥5 points above their previous PHQ-9 score or endorsement of the self-harm item) triggered further assessment. When elevated scores or suicide risk were identified, a psychologist from the study team (not blinded to allocation) conducted a risk assessment by telephone, and a management plan was implemented if required. These indicators were used to monitor participant safety but were not classified as adverse events. Participants could report concerns or adverse events at any time during the study by contacting the research team, as outlined in the participant information provided at trial commencement (passive surveillance).

### Randomization

Once baseline data were collected, participants were contacted by a member of the study team, who initiated randomization during the phone call using a central, computer-automated system. An allocation sequence with permuted block sizes of 2, 4, and 6 and a 1:1 allocation ratio was pregenerated by an independent IT professional using a computer-based random number generator. The allocation schedule was concealed from the project team. Allocation concealment was ensured through centralized, computer-automated administration. When the researcher indicated within the system that a participant was ready to be randomized, the system automatically assigned the participant to one of the 2 conditions. Study personnel enrolling participants did not have access to the allocation sequence. The allocation was then revealed to the participant. Following the phone call, participants allocated to DadBooster were emailed login details to access the program.

### Blinding

Given the nature of the intervention, participants were not blinded to treatment allocation after randomization. The intervention was delivered online, and no clinicians were involved in treatment delivery. However, outcomes assessment at the 12-week follow-up using the QuickSCID-5 was conducted by psychologists who were blinded to treatment allocation. Participants were instructed not to disclose their group allocation during these assessments to minimize the risk of unblinding. Data analysis was also conducted blind to allocation, with the dataset coded using nonidentifying group labels (A and B), and intervention-specific information removed prior to analysis. Although the trial registration was recorded as “Open (masking not used),” this reflected the absence of participant blinding and did not capture the blinding of outcome assessors and the data analyst.

### Sample Size

For the outcome measure of depressive symptom severity (DASS-21), data from a previous trial of online CBT for perinatal depression [46] provided relevant estimates of variability in baseline scores (mean 15.05=moderate range, SD 6.05 points). On this basis, a difference of 6 points would be necessary to move mean scores from the “moderate” to the “normal” category (normal range=0‐9 points) of depressive symptoms. We considered this to be the minimum clinically important difference in continuous scores of depression severity. With power of 0.8 at α=.05, the required n=15.7(6.05/6)^2^, which equals 16. Allowing for 25% attrition gives n=16/(1−0.25), which equals 21.28, and we rounded up to 25 per condition. Thus, we aimed for a total sample size of 50 to achieve sufficient power to detect a clinically important difference in the primary outcome measure of depressive symptom severity. No interim analyses were conducted or planned. The trial did not include an independent data monitoring committee. No formal stopping guidelines were specified, and the trial proceeded as planned to completion.

### Statistical Methods

The prespecified outcomes and analytical approach were documented in the trial registration record (ACTRN12622001029785). Although the registered analysis plan specified mixed-effects regression models for continuous outcomes, planned analyses of primary and secondary continuous outcomes (excluding PHQ-9) were changed prior to commencement of data analysis to analysis of covariance (ANCOVA), with baseline scores included as covariates. This approach was adopted because the primary comparison of interest was the prespecified 12-week primary end point. ANCOVA, adjusting for baseline scores, provides an efficient and statistically robust estimate of between-group differences at a single follow-up time point. Accordingly, the 9-week time point was not included in analyses, apart from the depression symptom trajectory (PHQ-9), which was analyzed using a mixed between-within ANOVA, with time point as a within-subjects factor and condition as a between-subjects factor.

The assumptions for each statistical test were tested and satisfied unless otherwise stated. Where assumptions of ANCOVA were violated (eg, homogeneity of regression slopes), difference scores were calculated (T3−T1), and a 2-tailed *t* test was conducted with the difference score as the dependent variable. A contingency table and Fisher exact test were used to assess changes in depression diagnosis (QuickSCID-5). No additional post-hoc analyses were conducted.

Primary and secondary outcomes were analyzed on an intention-to-treat basis, including all randomized participants analyzed in the groups to which they were assigned. Each analysis was conducted twice: once using complete case data and once after imputing missing values. Expectation maximization was used for imputation, as data were missing completely at random (the Little Missing Completely at Random test: *χ*^2^_87_=80.9; *P*=.66). Results are presented for the imputed dataset, which were quantitatively consistent with the complete case analyses (Multimedia Appendix 1), supporting the robustness of the results. α was set at .05. Effect sizes are reported as partial eta squared (η_p_^2^).

Descriptive statistics on complete case data are reported for program engagement, feedback and satisfaction, and use of other services. Data were analyzed using SPSS Statistics (version 22; IBM Corp).

### Ethical Considerations

This trial was approved by the human research ethics committee of Austin Health on August 31, 2021 (Project: HREC/73980/Austin-2021). All participants provided informed consent prior to participation in the study. Participant data were collected and stored in accordance with ethical guidelines, with all data deidentified prior to analysis to ensure confidentiality. Participants were reimbursed Aus $25 (Aus $1=US $0.71 as of August 18, 2026) for their time completing questionnaires at each of the 3 main time points: baseline, 9 weeks after enrollment, and 12 weeks after enrollment. No identifying information or images of participants have been included in this paper.

## Results

### Participant Flow and Recruitment

**Figure 2.**
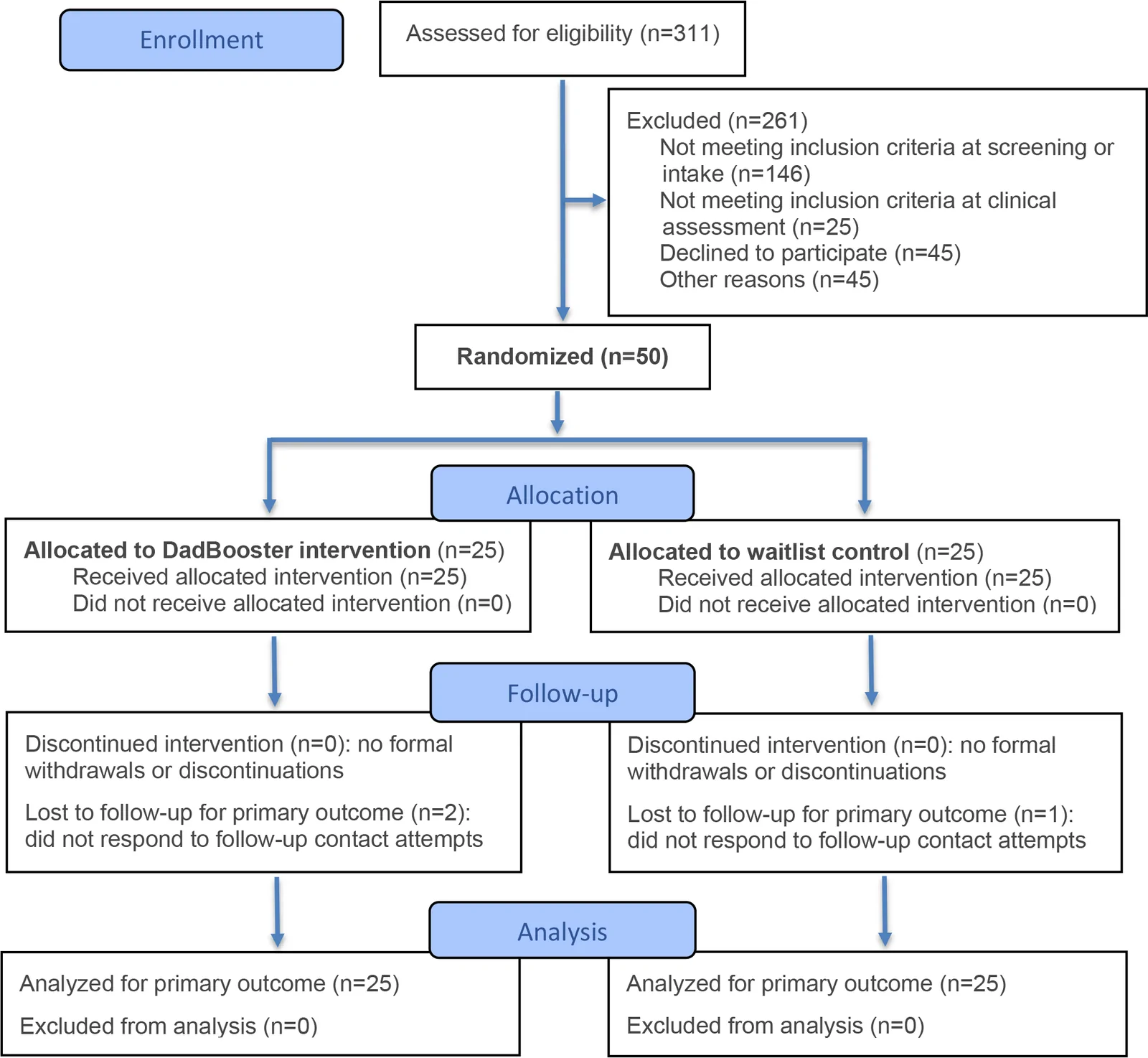
CONSORT (Consolidated Standards of Reporting Trials) flow diagram of participant progress through the phases of the randomized controlled trial of the DadBooster web-based, self-guided cognitive behavioral therapy program for fathers with postnatal depression (enrollment, allocation, follow-up, and data analysis). *Main reasons for exclusion: receiving therapy or antidepressant treatment (n=64), baby not <12 months (n=46), Patient Health Questionnaire-9<9 (n=38). Prospective participants may have met more than 1 exclusion criterion.

In total, 50 participants were recruited between August 2022 and July 2024 (DadBooster: n=25; waitlist control: n=25; Figure 2), with final follow-up in October 2024. Participants were followed for 12 weeks from enrollment. The trial was completed as planned, with recruitment ceasing once the target sample size was reached. Primary outcome data were collected from 94% (47/50) of participants. No adverse events were identified.

### Intervention and Comparator Delivery

The DadBooster intervention was delivered as intended via the online platform and automated SMSs, with no deviations in delivery. Participants completed sessions in a self-directed manner, with automated SMSs acting as reminders and engagement prompts.

All participants in the DadBooster condition (25/25) visited at least 1 session of the program, 80% (20/25) visited 4 or more sessions, and 52% (13/25) visited all 6 sessions. The mean number of sessions attended was 4.7 (SD 1.8). On average, users visited the program on 10 (SD 6.6; range 1‐25) days. The average total time spent using the program was 132 (SD 96.1; range 9‐358) minutes. On average, 3 of a possible 8 (SD 2.5; range 0‐8) library articles were accessed. The most popular article was “Managing your stress,” with 52% (13/25) of participants accessing this article. In total, 44% (11/25) of participants invited their partners to access the partner support website, and 32% (8/25) of partners accessed the website.

Participants allocated to the waitlist control condition did not receive access to the intervention during the study period and received usual care. The use of concomitant interventions was reported in both conditions. In total, 41% (9/22) of participants in the DadBooster condition reported accessing 1 or more additional sources of support during the trial, compared to 78% (18/23) of participants in the waitlist control (see Table 1 for a breakdown of support types).

**Table 1.** Sources of formal and informal support accessed by participants during the DadBooster trial, as reported at 12 weeks after enrollment^a^.

	DadBooster (n=22), n (%)	Waitlist control (n=23), n (%)
Psychologist or counselor	5 (23)	10 (40)
Medication for depression	0 (0)	3 (13)
Talked to a doctor who gave advice	3 (14)	9 (39)
Internet-based program other than DadBooster	0 (0)	1 (4)
Self-help book	3 (14)	4 (17)
Other (mental health-related podcasts and psychiatrist)	0 (0)	2 (8)

a Participants may have obtained support from more than 1 source.

### Baseline Data

Table 2 shows participant baseline characteristics. The groups appear comparable overall. As is appropriate in an RCT, no between-group significance tests were conducted on baseline variables [33].

**Table 2.** Baseline demographic characteristics of participants enrolled in the DadBooster trial (N=50).

	DadBooster (n=25)	Waitlist control (n=25)
Father’s age (years)		
Mean (SD)	35.7 (5.5)	34.2 (4.1)
Range	27‐50	24‐41
Baby’s age (months)		
Mean (SD)	4.8 (3.5)	3.8 (3.0)
Range	0‐11	0‐11
EPDS^a^ at screening		
Mean (SD)	16.2 (3.1)	17.4 (3.1)
Range	10-23	11-24
Diagnosis, n (%)		
Current major depression	25 (100)	21 (84)
Current depressive disorder with insufficient symptoms	0 (0)	4 (16)
Born in Australia, n (%)	22 (88)	20 (80)
Number of children (including recent baby), n (%)		
1	20 (80)	17 (68)
2	3 (12)	7 (28)
≥3	2 (8)	1 (4)
Married or living with partner, n (%)	25 (100)	25 (100)
Highest education, n (%)		
Apprenticeship or certificate level or less	5 (20)	6 (24)
Advanced diploma or diploma	4 (16)	5 (20)
Undergraduate degree	11 (44)	10 (40)
Postgraduate degree	5 (20)	4 (16)
Income, n (%)		
Up to Aus $80,000^b^	3 (12)	2 (8)
Greater than Aus $80,001^b^	22 (88)	21 (84)
Did not wish to divulge	0 (0)	2 (8)

a EPDS: Edinburgh Postnatal Depression Scale.

b Aus $1=US $0.71 as of August 18, 2026.

### Primary Depression Outcomes

#### Symptom Severity

After controlling for baseline DASS Depression scores, mean depression symptoms on the DASS Depression subscale at 12 weeks were significantly lower for the DadBooster condition than for the waitlist control condition (adjusted mean difference −7.26, 95% CI −11.34 to −3.18; *F*_1,47_=12.81; *P*<.001; η_p_^2^=0.21), indicating a very large effect size.

In the DadBooster condition, average scores on the DASS Depression subscale reduced from the high end of the “moderate” severity range (14-20) at baseline (mean 20.0, SD 7.1) to the “normal” range (0‐9) at 12 weeks (mean 5.7, SD 5.0). This represented an average 72% reduction in DASS Depression scores in the DadBooster condition, compared to a 40% reduction in the waitlist control condition (reduced from the “severe” range to “mild”; see Table 3 for means and SDs).

**Table 3.** Primary and secondary outcomes over time among participants in the DadBooster trial^a^.

	DadBooster (n=25), mean (SD)	Waitlist control (n=25), mean (SD)
Primary outcome		
Depression symptoms (DASS-21^b^)		
Baseline	20.0 (7.1)	23.0 (8.3)
12 weeks	5.7 (5.0)	13.9 (9.1)
Secondary outcomes		
Depression trajectory (PHQ-9^c^)		
Baseline	14.2 (3.7)	13.3 (3.2)
2 weeks	8.8 (3.8)	9.5 (5.0)
4 weeks	6.2 (2.8)	9.3 (5.0)
9 weeks	4.6 (2.9)	8.1 (4.0)
12 weeks	4.2 (3.5)	7.6 (5.2)
Stress (DASS-21)		
Baseline	21.9 (6.5)	21.8 (6.1)
12 weeks	10.7 (7.0)	17.2 (9.3)
Anxiety (DASS-21)		
Baseline	8.6 (6.7)	9.1 (8.4)
12 weeks	2.7 (3.2)	6.6 (8.7)
Negative thinking (ATQ^d^)		
Baseline	45.0 (20.0)	49.4 (24.1)
12 weeks	13.9 (12.0)	34.6 (31.5)
Behavioral activation (BADS^e^)		
Baseline	58.4 (18.7)	64.4 (18.0)
12 weeks	47.6 (14.7)	55.4 (21.3)
Parenting self-efficacy (PSOC^f^)		
Baseline	19.0 (6.6)	21.2 (6.5)
12 weeks	27.2 (6.7)	24.4 (5.8)
Relationship satisfaction (DAS-7^g^)		
Baseline	20.6 (5.2)	19.9 (5.5)
12 weeks	24.4 (4.6)	22.0 (6.6)

a Values are from intention-to-treat analyses after imputation of missing values.

b DASS-21: Depression Anxiety Stress Scale-21.

c PHQ-9: Patient Health Questionnaire-9.

d ATQ: Automatic Thoughts Questionnaire.

e BADS: Behavioral Activation for Depression Scale.

f PSOC: Parenting Sense of Competence Scale.

g DAS-7: Dyadic Adjustment Scale-7.

#### Clinical Diagnosis

A contingency table and Fisher exact test were used to assess changes in depression diagnosis (QuickSCID-5), as the assumption of minimum expected cell frequency for chi-square was violated. In the DadBooster condition, 2 of 23 (9%) participants assessed still met diagnostic criteria for a depressive disorder at 12 weeks compared with 7 of 24 (29%) participants assessed in the waitlist control condition. The intention-to-treat analysis found that the association between condition and diagnosis was not significant (DadBooster: 2/25; waitlist control: 7/25; Fisher exact test: *P*=.14).

### Secondary Outcomes

#### Trajectory of Depressive Symptoms

**Figure 3.**
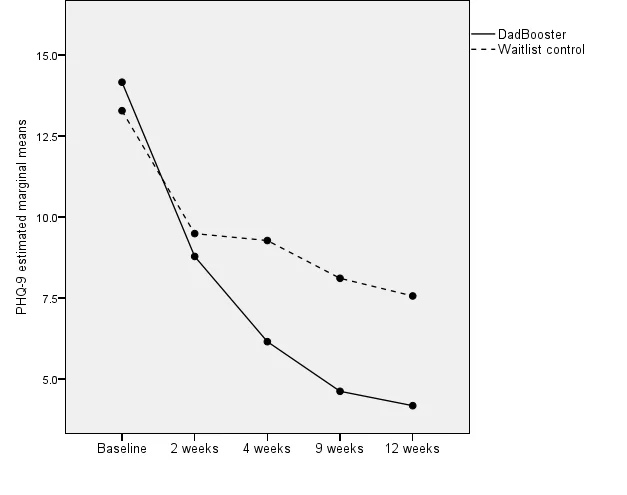
Trajectory of depressive symptoms (PHQ-9) scores from baseline to 12 weeks among participants in the DadBooster trial (intention-to-treat analysis with imputed missing data; n=25 per group). PHQ-9: Patient Health Questionnaire-9.

There was a significant interaction effect, indicating that the change in PHQ-9 scores over the course of the study time points was significantly different between the groups (Wilks Λ=0.74; *F*_4,45_=4.00; *P*=.007; η_p_^2^=0.26), representing a very large effect size (Figure 3). The main effect for time was also significant (Wilks Λ =0.22; *F*_4,45_=39.45; *P*<.001; η_p_^2^=0.78), indicating that there was a significant reduction in PHQ-9 scores over the 5 different time points. There was also a main effect for group (*F*_1,48_=4.62; *P*=.04; η_p_^2^=0.09), indicating that the 2 groups differed significantly in PHQ-9 scores.

#### Stress and Anxiety Symptom Severity

After controlling for baseline DASS Stress scores, mean stress symptoms on the DASS Stress subscale at 12 weeks in the DadBooster condition were significantly lower than that in the waitlist control condition (adjusted mean difference −6.55, 95% CI −11.01 to −2.09; *F*_1,47_=8.73; *P*=.005; η_p_^2^=0.16), indicating a large effect size. Severity scores were in the “moderate” range for both conditions at baseline and reduced to the “normal” range in the DadBooster condition compared to the “mild” range in the waitlist control condition.

As the ANCOVA assumption, homogeneity of regression slopes, was violated for the DASS Anxiety subscale, a 2-tailed *t* test of difference scores was conducted. The change in anxiety symptoms over the study was not significantly different between the conditions (mean difference −3.40, 95% CI −7.03 to 0.23; *t*_48_=−1.88; *P*=.07). Scores in the DadBooster condition decreased, on average, by 5.9 (SD 6.2) points, while scores in the waitlist control condition decreased, on average, by 2.6 (SD 6.6) points.

#### Negative Thinking and Behavioral Activation

After controlling for baseline negative thinking (Automatic Thoughts Questionnaire) scores, mean negative thinking scores at 12 weeks in the DadBooster condition were significantly lower than that of the waitlist control condition (adjusted mean difference −18.10, 95% CI −29.79 to −6.42; *F*_1,47_=9.71; *P*=.003; η_p_^2^=0.17), indicating a large effect size. After controlling for baseline behavioral activation (Behavioral Activation for Depression Scale) scores, mean behavioral activation scores at 12 weeks in the DadBooster condition were not significantly different than that of the waitlist control condition (adjusted mean difference −4.72, 95% CI −13.91 to 4.46; *F*_1,47_=1.07; *P*=.31; η_p_^2^=0.02).

#### Parenting Self-Efficacy and Relationship With Partner

After controlling for baseline parenting self-efficacy (Parenting Sense of Competence Scale) scores, mean parenting self-efficacy scores at 12 weeks in the DadBooster condition were significantly higher than that of the waitlist control condition (adjusted mean difference 4.06, 95% CI 1.16-6.95; *F*_1,47_=7.93; *P*=.007; η_p_^2^=0.14), indicating a large effect size. After controlling for baseline relationship satisfaction (DAS-7) scores, mean relationship satisfaction scores at 12 weeks in the DadBooster condition were not significantly different than those of the waitlist control condition (adjusted mean difference 1.92, 95% CI −0.41 to 4.25; *F*_1,47_=2.74; *P*=.11; η_p_^2^=0.06).

#### Program Feedback and Satisfaction

In total, 96% (21/22) of DadBooster participants rated the program overall as “somewhat helpful” to “very helpful.” Participants were most satisfied with the strategies for reducing negative thinking and increasing positive thinking (see Table 4 for a breakdown of satisfaction with program features). Average scores on the System Usability Scale indicated acceptable usability of the DadBooster program (mean 71, SD 16).

**Table 4.** Satisfaction ratings (“moderately satisfied” or “very satisfied”) for each key feature of the DadBooster program from participants in the DadBooster trial^a^.

	Rated satisfaction as “moderately satisfied” or “very satisfied” (n=22), n (%)
Strategies for reducing negative thinking	15 (68)
Strategies for increasing positive thinking	14 (64)
Pleasant activities	13 (59)
Text messages	12 (55)
Mood tracking	11 (50)
Library articles	11 (50)
Videos	9 (41)
Partner support program	8 (36)

a Satisfaction response options were 1=not at all satisfied, 2=somewhat satisfied, 3=moderately satisfied, 4=very satisfied.

Participants were interested in additional topics for library articles, with “Feeling angry and irritable” being the most requested (13/22, 59%), followed by “Men’s attitudes to seeking help” (10/22, 45%) and “Alcohol and substance use” (4/22, 18%). No other topics were suggested by participants.

## Discussion

### Principal Findings

Postnatal depression affects at least 1 in 10 fathers [3] and negatively impacts the whole family system [47]. Many fathers with postnatal depression do not seek help or engage with effective treatment [17]. Digital treatment offers many advantages compared to traditional options that may appeal to fathers, such as privacy, accessibility, and convenience [16,17]. This RCT evaluated the efficacy of a new online treatment for postnatal depression in fathers, DadBooster. To our knowledge, this is the first online treatment designed to treat clinically diagnosed postnatal depression in fathers [22]. The results showed that DadBooster is an effective treatment for postnatal depression, with fathers in the DadBooster condition experiencing a reduction in symptom severity of 72% on average, significantly greater than in the waitlist control condition. Fathers who received DadBooster also showed significantly greater improvements in stress, parenting self-efficacy, and negative automatic thoughts. There were no significant differences between the conditions in the proportion of fathers still meeting diagnostic criteria at 12 weeks, or in anxiety, behavioral activation, and partner relationship quality. The program was well-accepted by fathers, and the number of registrations to the trial indicated good interest in the program among fathers.

### Effects on Postnatal Depression in Fathers

#### Symptom Severity

The results were consistent with findings from our previous trials of MumMoodBooster [28,31]—the treatment for postnatal depression in mothers that inspired DadBooster. Participants in the DadBooster condition showed a significantly larger reduction in depression symptoms at 12 weeks compared to the waitlist control condition, with average symptom levels in the DadBooster condition reducing to the “normal” range [40]. Importantly, these results stand in contrast to the only published RCT of a digital intervention (mindfulness app) for postnatal depression in fathers [29], which reported no between-group differences in depression, anxiety, or stress, possibly due to low adherence to the intervention and moderate attrition over the study.

Visual inspection of the symptom trajectories suggests an initial reduction in depression symptoms in both conditions during the first few weeks following enrollment. However, the DadBooster condition appeared to show a more pronounced decline in symptoms over the remainder of the study, with a pattern consistent with continued improvement compared to the waitlist control. The initial reductions observed in both conditions may reflect potential benefits of the intake and assessment procedure, which included telephone contact, assessment and diagnosis with a psychologist, and a referral letter sent to each participant’s general practitioner. A recent meta-analysis showed that contact with a health professional prior to commencing an online treatment for depression (such as a diagnostic interview) is associated with better treatment outcomes [48].

#### Clinical Diagnosis

A notable strength of this study was the inclusion of fathers who met clinical diagnostic criteria for depression. Several reviews have noted the lack of studies with men experiencing clinically significant depression [20,22], which has limited research on the suitability of interventions for this population. The only published trial of a digital intervention targeting postnatal depression in fathers [29] recruited a screened sample rather than requiring a formal clinical diagnosis for inclusion. In the present study, while severity of depression symptoms was significantly reduced following DadBooster, the between-group difference in remission rates did not reach statistical significance. However, the observed proportion of fathers still meeting diagnostic criteria for depression in DadBooster compared to waitlist control suggests a potential difference, consistent with the results observed for symptom severity. The lack of statistical significance may reflect the modest sample size and the additional support accessed by the waitlist control condition during the study period. More than three-quarters of the participants in the waitlist control condition sought additional support, most commonly from a health professional. In contrast, less than half of the fathers in the DadBooster condition sought additional support. This higher level of use in the waitlist control condition may have reduced between-group differences and thus provided a more conservative test of the intervention’s effects.

#### Effects on Stress and Anxiety

Consistent with findings from MumMoodBooster trials [28,31], fathers in the DadBooster condition demonstrated significantly greater reductions in stress compared to fathers in the waitlist control. However, no significant differences were observed for anxiety. Although depression and anxiety are highly comorbid [49], mean baseline anxiety scores were relatively low in both groups, falling within the “mild” range [40], which likely limited the scope for detectable improvement. In contrast, baseline stress scores were within the “moderate” range [40] for both groups, consistent with systematic review findings that fathers experience heightened stress during the perinatal period, contributing to mental health difficulties, such as depression [50]. Further, previous research suggests that men who strongly adhere to traditional masculine norms may conceptualize and describe their depression as “stress” [21]. Thus, reductions in perceived stress may represent an important and meaningful treatment outcome for fathers experiencing depression. The salience of stress among participating fathers may also be reflected in the program engagement data, with the “Managing your stress” article being the most frequently viewed resource in the DadBooster library.

#### CBT Skill Acquisition

Significant between-group differences were observed for automatic thoughts but not for behavioral activation. One possible explanation is that the competing demands of work, infant care, and partner support leave fathers with little time for self-directed activities [51], making behavioral activation strategies more challenging to implement. We can also speculate that men’s attitudes and traditional societal masculine norms—for example, expectations to be the provider, remain strong, prioritize others’ needs before their own, and support their partner—may have been a barrier to engaging in pleasant activities, consistent with literature on the association between adherence to masculine norms and men’s well-being behaviors [21]. It is possible that guilt associated with taking time out may have reduced engagement with the behavioral activation component of DadBooster. Research on CBT for men has highlighted the importance of addressing both cognitions and behaviors associated with traditional masculine norms when treating depression [52]. The findings of the present study may provide some insight into components of treatment specific to men that have been called for in previous reviews [20,52].

#### Parenting Self-Efficacy

Importantly, significant between-group differences were found for parenting self-efficacy. This finding is noteworthy because a parent’s confidence in their ability to parent their child is strongly linked to more positive parent-infant interactions and improved child developmental outcomes [53]. The negative effects of postnatal depression in fathers on child outcomes are well-documented [14]. By strengthening parenting self-efficacy, the program may have broader implications for child outcomes, although this remains to be explored in future studies.

#### Partner Relationship

Consistent with MumMoodBooster trials [28,31], no significant between-group differences were observed in fathers’ relationships with their partners. Baseline scores for both conditions were within the “distressed” range. Elevated levels of relationship distress in this sample are consistent with previous research identifying relationship difficulties as a key challenge during the transition to fatherhood that can impact paternal mood [51]. Although some improvement in the couple relationship was noted in the DadBooster condition, average scores increased to just above the distressed threshold at 12 weeks [43]. However, less than half of the participants invited their partners to access the partner support website, and only one-third of participants’ partners accessed it. This may reflect the level of relationship distress and could have limited any potential benefits to their partners’ mental health and the couple relationship. It may also indicate adherence to traditional gender norms [20], where fathers feel the need to be strong and not burden their partners at a time when they may also be struggling. A proportion of partners may also have been experiencing their own mental health difficulties, given the established association between paternal and maternal depression in the postnatal period [13]. Future research could explore strategies to increase engagement with the partner support website, which also has links to support for postnatal depression in mothers, and could consider incorporating additional relationship-focused resources into the program.

#### Engagement and Satisfaction With DadBooster

Fathers in the DadBooster condition demonstrated high levels of engagement with the program, with most visiting 4 or more of the 6 sessions and, on average, visiting the program on several days. This level of program use is encouraging, as dropout rates tend to be higher in self-guided interventions than in guided interventions [54], and men are more likely to drop out [55]. The recent trial by Teague et al [29] reported low adherence among fathers to their mindfulness app and moderate attrition. It is possible that, in the present study, the intake and assessment calls with a psychologist may have been helpful for enhancing engagement with the DadBooster intervention, consistent with meta-analytic results showing that contact (eg, a diagnostic interview) prior to commencing online treatment is associated with greater adherence with the intervention [48]. Satisfaction with the program was also favorable, with almost all fathers indicating that they found the program to be “somewhat” to “very” helpful.

### Strengths and Limitations

This study had several strengths, including the use of a clinically diagnosed sample, blinded assessments and intention-to-treat analyses, very low attrition, and rigorous conduct in adherence to CONSORT guidelines [33]. However, limitations should also be acknowledged. As with most RCTs, the generalizability of these findings warrants consideration, as the sample comprised only fathers who were willing to participate in research and be randomized to either condition. Furthermore, fathers with comorbid mental health conditions or who were not English-speaking were excluded, so no information is available about applicability to more diverse populations. In real-world settings, DadBooster is likely to be accessed by a more heterogenous group of fathers, including those with subthreshold symptoms or a primary anxiety presentation. Given that CBT targets transdiagnostic cognitive and behavioral processes across a range of depression and anxiety presentations [56], it is possible that the program may provide benefit across a continuum of symptom severity; however, this remains to be established in real-world use. In addition, the high adherence observed in the present RCT may not be replicated in real-world use. The relatively short period of follow-up (12 weeks) also restricts conclusions regarding the longer-term maintenance of symptom improvements.

### Future Research

Future research should examine the longer-term maintenance of symptom improvements, as well as engagement and effectiveness in real-world samples that include a broader range of symptom severities and presentations. As DadBooster is currently being rolled out nationally with Australian government funding, future evaluations will be able to examine uptake, engagement, and outcomes in a real-world setting. While DadBooster was developed as a treatment for fathers with postnatal depression, preventative approaches for fathers at risk may also be valuable. Accordingly, future work could explore whether similar interventions can be adapted for earlier preventative use to reduce the onset of postnatal depression. Additional research may also examine potential impacts on child outcomes and identify strategies to enhance engagement with the partner support website.

### Conclusions

This RCT provides evidence for the efficacy of DadBooster, the first online treatment specifically for postnatal depression in clinically depressed fathers. This addresses a critical gap in father-specific treatment approaches for postnatal depression [22], an area in which research has until recently focused primarily on mothers. DadBooster improved depression symptoms, stress, parenting self-efficacy, and negative automatic thoughts, and was well-accepted by fathers. By providing evidence for an intervention specifically designed for fathers with potential for broader scalability, this study advances the limited literature on treatment approaches for postnatal depression in fathers. Given its accessibility, privacy, and convenience, DadBooster is a promising and potentially impactful intervention for supporting fathers.

## Supplementary material

10.2196/94541 Multimedia Appendix 1 Supplementary results from complete-case analyses. Primary and secondary outcome results based on complete-case analyses of randomized controlled trial participants with available data.

10.2196/94541 Checklist 1 CONSORT-EHEALTH checklist.

10.2196/94541 Checklist 2 CONSORT 2025 checklist.

## Abbreviations

ANCOVA: analysis of covariance
CBT: cognitive behavioral therapy
CONSORT: Consolidated Standards of Reporting Trials
CONSORT-eHEALTH: Consolidated Standards of Reporting Trials of Electronic and Mobile Health Applications and Online Telehealth
DAS-7: Dyadic Adjustment Scale-7
DASS: Depression Anxiety Stress Scale
DASS-21: Depression Anxiety Stress Scale-21
DSM-5: Diagnostic and Statistical Manual of Mental Disorders-5
PHQ-9: Patient Health Questionnaire-9
QuickSCID-5: Quick Structured Clinical Interview for DSM-5
RCT: randomized controlled trial
