# Supplementary material for: Internet-Delivered Cognitive Behavioral Therapy for Fathers With Postnatal Depression: Randomized Controlled Trial of DadBooster
Source: J Med Internet Res. 2026 Sep 1;28:e94541. doi: 10.2196/94541 (PMC13532688; doi:10.2196/94541)
Supplement: Supplementary file 2 [file jmir-v28-e94541-s001.docx]

**Table S1.** Primary and secondary outcomes from complete case analyses

|  |  | **DadBooster**  ***M* (*SD*)** | **Waitlist Control**  ***M* (*SD*)** | **Test** | ***df*** | ***P*** | **Effect size** |
| --- | --- | --- | --- | --- | --- | --- | --- |
| **Depression symptoms (DASS-21)** | |  |  |  |  |  |  |
|  | Baseline | 20.0 (7.1) *n*=25 | 23.0 (8.3) *n*=25 |  |  |  |  |
|  | 12-weeks | 5.0 (4.7) *n*=23 | 14.2 (9.2) *n*=24 | *F*=14.75 | 1, 44 | <.001 | η_p_^2^=.25 |
| **Depression diagnosis (QuickSCID-5)** | |  |  | Fisher’s exact test |  | .14 |  |
| **Depression trajectory  (PHQ-9)** | |  |  |  |  |  |  |
|  | Baseline | 14.2 (3.7) *n*=25 | 13.3 (3.2) *n*=25 |  |  |  |  |
|  | 2-weeks | 8.7 (3.9) *n*=23 | 9.9 (5.0) *n*=23 |  |  |  |  |
|  | 4-weeks | 5.6 (2.6) *n*=22 | 9.8 (5.1) *n*=21 |  |  |  |  |
|  | 9-weeks | 4.3 (2.8) *n*=22 | 8.6 (4.1) *n*=20 |  |  |  |  |
|  | 12-weeks | 3.8 (3.4) *n*=23 | 7.7 (5.3) *n*=24 |  |  |  |  |
|  | Interaction |  |  | Wilks’ Lambda=.72, *F*=3.61 | 4, 37 | .014 | η_p_^2^=.28 |
|  | Time |  |  | Wilks’ Lambda=.22, *F*=33.82 | 4, 37 | .001 | η_p_^2^=.79 |
|  | Group |  |  | *F*=8.86 | 1, 40 | .005 | η_p_^2^=.18 |
| **Stress (DASS-21)** | |  |  |  |  |  |  |
|  | Baseline | 21.9 (6.5) *n*=25 | 21.8 (6.1) *n*=25 |  |  |  |  |
|  | 12-weeks | 10.1 (6.8) *n*=23 | 17.3 (9.5) *n*=24 | *F*=9.51 | 1, 44 | .004 | η_p_^2^=.18 |
| **Anxiety (DASS-21)** | |  |  |  |  |  |  |
|  | Baseline | 8.6 (6.7) *n*=25 | 9.1 (8.4) *n*=25 |  |  |  |  |
|  | 12-weeks | 2.3 (3.1) *n*=23 | 6.7 (8.9) *n*=24 | *t*=-1.61 | 45 | .11 |  |
| **Negative thinking (ATQ)** | |  |  |  |  |  |  |
|  | Baseline | 45.0 (20.0) *n*=25 | 49.4 (24.1) *n*=25 |  |  |  |  |
|  | 12-weeks | 12.1 (10.8) *n*=22 | 37.3 (31.3) *n*=23 | *F*=11.70 | 1, 42 | .001 | η_p_^2^=.22 |
| **Behavioural activation (BADS)** | |  |  |  |  |  |  |
|  | Baseline | 58.4 (18.7) *n*=25 | 64.4 (18.0) *n*=25 |  |  |  |  |
|  | 12-weeks | 46.6 (15.3) *n*=22 | 57.0 (21.5) *n*=23 | *F*=1.38 | 1, 42 | .25 | η_p_^2^=.03 |
| **Parenting self-efficacy (PSOC)** | |  |  |  |  |  |  |
|  | Baseline | 19.0 (6.6) *n*=25 | 21.2 (6.5) *n*=25 |  |  |  |  |
|  | 12-weeks | 28.0 (6.6) *n*=22 | 24.0 (5.8) *n*=23 | *F*=11.92 | 1, 42 | .001 | η_p_^2^=.22 |
| **Relationship satisfaction (DAS-7)** | |  |  |  |  |  |  |
|  | Baseline | 20.6 (5.2) *n*=25 | 19.9 (5.5) *n*=25 |  |  |  |  |
|  | 12-weeks | 24.6 (4.8) *n*=22 | 21.6 (6.7) *n*=23 | *F*=3.26 | 1, 42 | .08 | η_p_^2^=.07 |

*M* = Mean. *SD* = Standard deviation. df = Degrees of freedom. η_p_^2^ = Partial eta squared. DASS-21 = Depression Anxiety Stress Scales. QuickSCID-5 = Quick Structured Clinical Interview for the DSM-5. PHQ-9 = Patient Health Questionnaire. ATQ = Automatic Thoughts Questionnaire. BADS = Behavioral Activation for Depression Scale. PSOC = Parenting Sense of Competence Scale. DAS-7 = Dyadic Adjustment Scale.
